# Cariprazine efficacy in a 40-year untreated case of a woman with predominantly negative symptoms of psychosis: A case report

**DOI:** 10.1192/j.eurpsy.2024.1568

**Published:** 2024-08-27

**Authors:** A. Mara, S. Myriknas

**Affiliations:** Psychiatric Hospital, Thessaloniki, Greece

## Abstract

**Introduction:**

Several studies have demonstrated the unfavorable and neurotoxic effects of untreated psychosis (UP) on the brain. An estimated 10 to 12 cc of brain tissue could be potentially damaged due to neuroinflammation and oxidative stress when a first episode of psychosis goes untreated. Other studies have found a correlation between the duration of untreated psychosis (DUP) and treatment resistance or nonresponse. Evidence-based schizophrenia treatment mainly relies upon the use of first and second-generation antipsychotics, without solid evidence that the former is superior to the latter regarding the treatment of negative symptoms. Both groups, however, can come with a risk of side effects. Cariprazine, a third-generation antipsychotic, represents a safe and effective treatment, targeting both the positive and negative symptoms of schizophrenia.

**Objectives:**

To report a clinical case of a woman with an extreme DUP with predominantly negative symptoms of schizophrenia and highlight the favorable outcome cariprazine monotherapy had on her global functioning.

**Methods:**

We report a clinical case of a 58-year-old woman with a history of a 40-year UP successfully treated with 4,5mg of cariprazine. The woman was brought involuntarily for psychiatric assessment at the emergency department with a clinical image of catatonic stupor and predominantly negative symptoms of psychosis. Her total PANSS score at admission was 129. The negative subscale score was 49. She was initially treated with 3mg cariprazine and 10mg olanzapine and was gradually left on 4,5mg cariprazine monotherapy with an adjunctive 30mg mirtazapine.

**Results:**

The patient was dismissed after 47 days of hospitalization. Cariprazine was effective in targeting both the cognitive and affective symptoms of long-standing UP. In the long-term, cariprazine also improved remnant delusional ideas of somatic and persecutory types, enhancing the patient’s social life, ensuring her support network, and assisting her integration into the community. The patient did not report any side effects, and her blood test results were within the normal range.

**Conclusions:**

Not all cases of schizophrenia are dramatic at presentation - some can have a chronic and insidious course predominated by negative symptoms. UP can lead to disastrous consequences for the patient’s biopsychosocial well-being, leading to future treatment resistance and disability. Although such cases of untreated psychosis seem to be from the past, we should be conscious of their existence and treat them with a patient-personalized and symptom-centered approach. Cariprazine was successful and effective in treating this patient with a remarkable course of UP.

**Disclosure of Interest:**

None Declared

